# Electrocatalytic Behavior and Determination of Amitriptyline Drug with MWCNT@Celllulose Composite Modified Glassy Carbon Electrode

**DOI:** 10.3390/ma13071708

**Published:** 2020-04-06

**Authors:** Aftab Aslam Parwaz Khan

**Affiliations:** Chemistry Department, Faculty of Science, King Abdulaziz University, P.O. Box 80203, Jeddah 21589, Saudi Arabia; aapkhan@kau.edu.sa

**Keywords:** cellulose, MWCNT, amitriptyline, electrochemical sensor

## Abstract

A novel nanocomposite of cellulose based on multiwalled carbon nanotube (MWCNT) was synthesized by a simple solution mixing–evaporation method. The morphology, thermal investigations, electrocatalytic oxidation of amitriptyline were analyzed at multi-walled carbon/cellulose nanocomposite in detail. The amitriptyline (AMT) drug was electrochemically studied in a phosphate buffer at different pH using the MWCNT/cellulose modified glassy carbon electrode (GCE). As per the linear relationship among AMT along with peak current, differential pulse voltammetry technique has been established for their quantitative pharmaceutical’s determination. The oxidation potential shifted negatively compared to GCE, showing that the MWCNT/cellulose modified electrode had an excellent catalytic activity for the AMT oxidation. The anodic peak current varied linear response with AMT’s concentration in the range of 0.5 to 20.0 μM with a LOD of 0.0845 μM and LOQ of 0.282 μM, respectively. The proposed method was effectively put on the determination of AMT in pharmaceutical and urine samples. This novel methodology is presented here as an example of a complete development methodology for the determination of amitriptyline drug and sensor for use in healthcare fields.

## 1. Introduction

Cellulose is considered the most abundantly produced, as well as extensively degraded, polymer in our environment and for harbors one-half of all organic carbon [1]. Cellulose is a highly crystalline, as well as a high-molecular weight polymer, that is insoluble and infusible in aqueous solvents. Carboxymethyl cellulose (CMC) is one of the essential industrial biopolymers. CMC is a semi-synthetic cellulose’s derivation created by partial substitution of the cellulose’s hydroxyl group by a far more appropriate carboxymethyl group as compared to cellulose [2,3]. CMC is a particularly derivative of cellulose having a carboxymethyl group that is bound to several hydroxyl organizations. The polar carboxymethyl group helps to make cellulose chemically reactive as well as soluble [4,5]. Wang et al. fabricated a graphene/CMC aerogel having more higher elasticity along with excellent conductivity materials by using a simple solution mixing method [6]. Haijian et al. made a super elastic nanotube of CMC/carbon composite aerogel by mixing unmodified multi-walled as well as single-walled carbon nanotubes in CMC aqueous solution along with directional freeze-drying technique [7].

The Carbon nanotubes (CNTs) possess sizeable specific surface area, hydrophilicity, and excellent water dispersibility [8]. So, CNTs are the most appropriate ideal inorganic additives to improve cellulose materials. CNTs consist of a graphite sheet role in the appearance of the cylinder. Carbon nanotubes have received full attention in electrochemistry and numerous areas covered in chemistry and physics due to the novel structural form like chemical, electrical, and mechanical properties [9]. Carbon nanotubes can encourage charge transfer reactions in electrode preparation [10]. The modification of electrode by multiwalled carbon nanotube (MWCNTs) designed for examination during sensing scheme has been predictable in the direction of outcome in low detection limit, maximum feeling like sensitivity, resistance to exterior outside fouling, and MWCNTs have been introduced the same as electrocatalysis. Several carbon nanotube customized electrodes have been done, as well as carbon nanotubes, paste electrodes, graphite electrodes intercalated through carbon nanotubes, and carbon nanotubes scattering, shed electrodes, etc. [11]. Throughout the previous years, several papers were reported on the growth as well as the application of cellulose/multi-walled carbon nanotube (MWCNT) composite in the fields of biomaterials, electrochemistry, biosensors [12,13,14,15,16], chemical vapor sensor as an ideal solution [17,18], cellulose with the help of a tetrabutylammonium acetate/DMSO have done the formation of regenerated cellulose fiber [19], composite fibers regenerated of MWCNT/cellulose dispersed in ammonium/DMSO mixed solvent [20] and nanocellulose attracted interest in energy storage research in LIB electrodes [21]. For dispersing Carbon nanomaterials in an aqueous medium having no chemical functionalization or maybe the inclusion of surfactant/water-soluble polymers [22,23]. By using the benefits, cellulose has been utilized as a binding for producing CNT electrodes through the aqueous solution procedure. Nevertheless, many electrodes have been made by mixing cellulose materials, fibril foam, like bacterial cellulose as well as cellulose nanofibril as a result of their interwoven web-like framework [24,25,26].

Molecules/ions are amphiphilic in aqueous solutions typically bring together at interfaces as well as self-concerted effort to appropriate their polar areas from contact having an aqueous stage [27,28,29]. A considerable number of drugs to perform like surfactants as well as phenothiazine derivatives, viz diphenylmethane, diphenhydramine chlorpromazine, tricyclic antidepressants, and amitriptyline [30].

Amitriptyline 3-(10,11-dihydro-5H-dibenzo[a,d]cycloheptene-5-ylidene)-N,N-dimethylpropan-1-amine amine is a tricyclic antidepressant agent that also has analgesic properties”. It is analgesic possessions concurrent to its humor changing goings-on/attributable to distinct pharmacological deed is unknown. Clinical trials show that oral amitriptyline (AMT) achieves at least a high-quality/thoughtful reply in up to 2/3 thirds of patients employing postherpetic neuralgia and other patients with painful diabetic neuropathy, neurogenic pain conditions which are commonly unfeeling to narcotic analgesics. AMT is an efficient drug with diarrhea leading irritable bowel syndrome (IBS). Some investigate the efficiency of AMT has been full of loopholes for a reason that of being short of control groups/failure to verify the analysis of IBS by ruling out organic causes.

In some cases, AMT has been used in grouping with other molecules, assembly it hard to be familiar with the clinical input of AMT [31]. It has been reported in the study that the combination of cellulose nanocrystals and multiwall carbon nanotubes has done effectively discriminate against the enantiomers of tryptophan and captopril [32,33]. Much research based on the cellulose nanocomposites have been executed to the reliable with various approaches [34,35,36,37,38,39,40,41]. Given the prospective pharmaceutical significance of AMT and the literature, a survey has revealed that no attention has been paid towards the electrochemical behavior of AMT with a detailed experimental study of the reaction becomes significant.

## 2. Experimental

### 2.1. Materials and Methods

Carboxymethyl cellulose (Fluka) was used without further purification. (CH_3_)_2_CO and CH_3_OH, K_3_Fe(CN)_6,_ and alumina slurry were used analytical and without further purification. AMT, Glutaraldehyde, along all other reagents have been analytical grades as well as obtained from Merck chemical company (Mumbai, Maharashtra, India). An AMT stock solution has been ready in double-distilled water.

### 2.2. Preparation of MWCNT/Cellulose Composites

Carboxymethyl cellulose/MWCNT nanocomposite was prepared by a simple solution mixing-evaporation method. In a typical procedure, 10 mg of MWCNT (purity >95%, diameter 20–45 nm, length several microns, surface area >500 m^2^/gm, synthesis by CVD%, Applied science innovations, Pune, Maharashtra, India India) has been sonicated in double-distilled water approximately for 2 h. A CMC sample of 0.99 g has been mixed in de-ionized water, having constant stirring to dissolve the CMC completely. After that CMC solution mixture has been slowly mixed with a well-dispersed MWCNT solution having sonication continuation. After the sonication of 3 h with the help of ice, the beaker of the reaction mixture has been put on a magnetic stirrer having continuous stirring at ambient temperature for a whole night. The precipitate has been filtered off, and after that, washed clearly with ethanol and distilled water, and subsequently freeze-dried. Then, 10 mg of any cellulose/MWCNT was dispersed in 10 mL of de-ionized water under sonication; the resulting composite suspension was reduced with hydrazine. At the end of the reduction, the suspensions have been filtered as well as washed by using (CH3)_2_CO, water, and CH_3_OH to remove the impurities. After that, the product was placed in a freezer for dried out. The benefits of this specific synthetic procedure are (1) this particular synthetic procedure doesn’t include some dangerous chemicals, moreover (2) this procedure is straightforward as well as affordable.

### 2.3. Fabrication of Electrode with GCE and MWCNT/Cellulose Composite 

Firstly, the electrode was made by polishing 3 mm diameter of GCE having alumina slurry (0.05 μm), smooth-wiped on a velvet pad. Aftwewards, for 20 min, the electrode was ultrasonicated and washed properly by using distilled water at room temperature. After drying out, MWCNT/cellulose composite dispersion (8 μL) decreased cast on the GCE as well as at room temperature; it was permitted to dry out for 1 h. Finally, a 2 μL of a glutaraldehyde aqueous solution (2%) was drop cast to crosslink the MWCNT/cellulose composite modified electrode the solidly as well as held to dry out for approximately 30 min. Furthermore, for removing non-bounded glutaraldehyde, the electrode was added to distilled water for near about 2 min. So, after that, the electrode was allowed to dry out at room temperature as well as put in the refrigerator until estimations had been taken.

### 2.4. Preparation of Human Urinal Samples

The urine samples were diluted 100 times with the PBS solution (pH = 7) before analysis without further pretreatments. A quantitative determination can be carried out by adding the standard solution of AMT into the detection system of a urine sample. DPV was recorded under optimized conditions.

### 2.5. Characterization of MWCNT/Cellulose Nanocomposite

The Carboxymethyl cellulose/MWCNT nanocomposite morphology has been obtained by using a JEOL organization Field Emission Scanning Electron Microscope (JSM-7600F, JEOL, Akishima, Japan) at various amplifications. DTG, as well as TGA sample, had been performed by Shimadzu DT-60 (10–9500 °C) a heating rate as well as airflow of 10.00 Cmin^−1^ along with 00.0 mL min^−1^, respectively. Potentiostat has been used for electrochemical experiments. For this experiment, Ag/AgCl, Pt wire, as well as electrode GCE manufactured by MWCNT/cellulose composites, had been utilized. After that, the electrode has been appropriately washed by using Labman, ultrasonic LMUC.

## 3. Results and Discussions

### 3.1. Study of Morphological and Thermal Analysis

**Figure 1:**
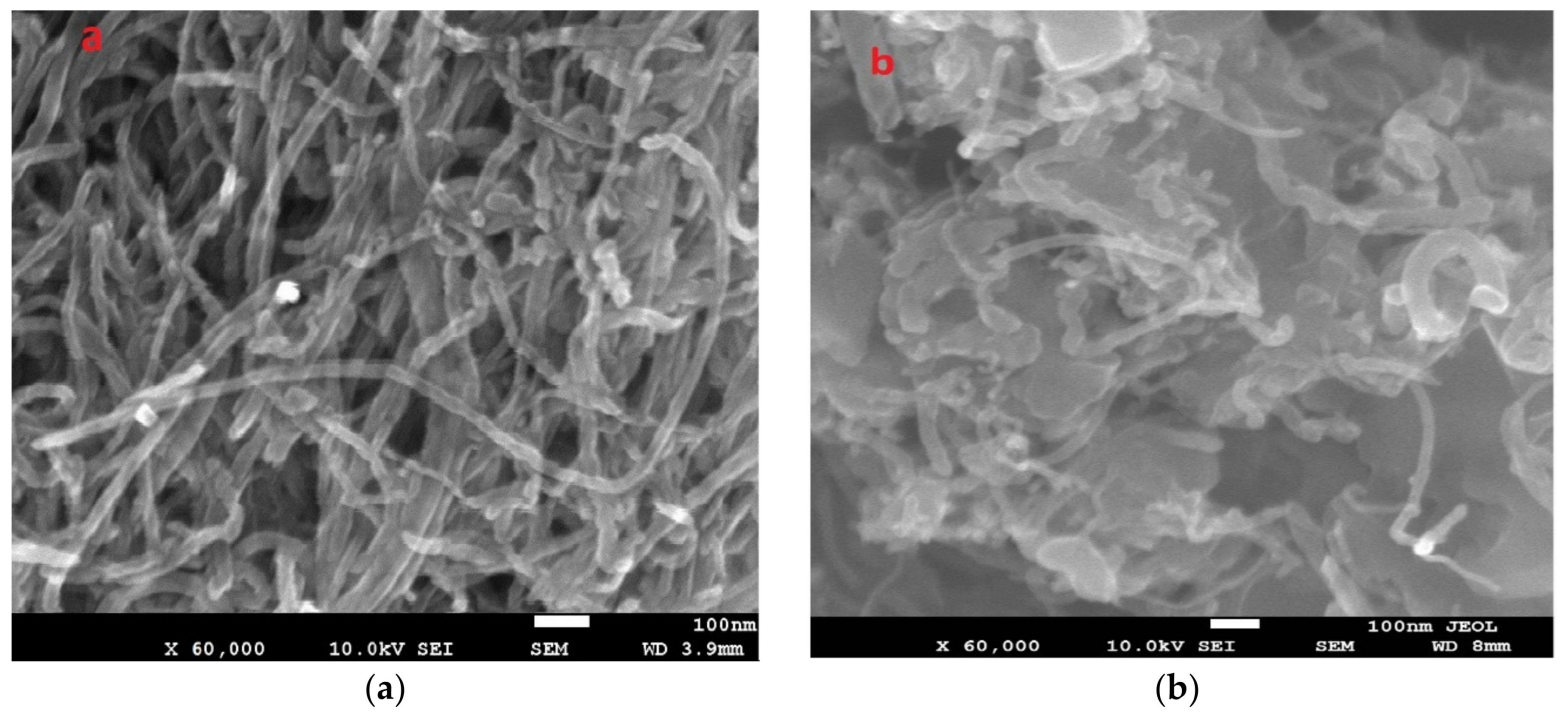
FE-SEM images of the (**a**) multiwalled carbon nanotube (MWCNT) (**b**) MWCNT/cellulose nanocomposite at the same magnifications prepared via simple solution mixing-evaporation method.

**Figure 2:**
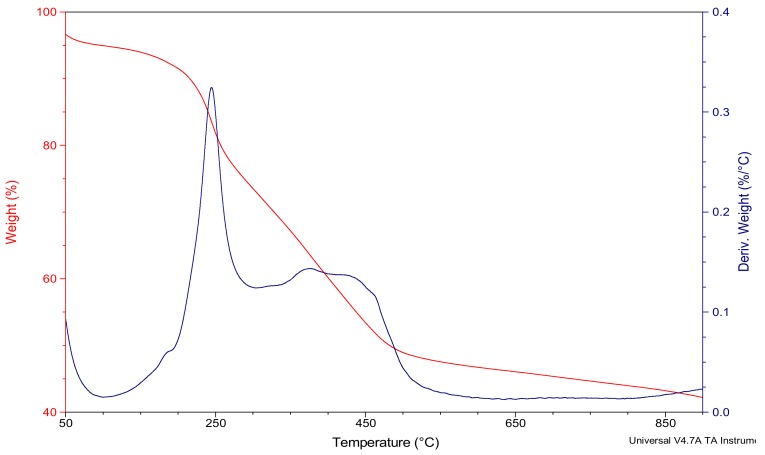
TGA spectra of MWCNT@celllulose nanocomposite showing degradation procedures.

SEM analysis is a well-defined method for the morphology research of as-synthesized materials, as well as it also provides essential information about the size, shape, and growth of many materials. MWCNTs, MWCNT/CMC composites, along with FE-SEM images, are shown in Figure 1. The FE-SEM images of the MWCNTs demonstrate a smooth surface and denser network without micropores in Figure 1a. However, Figure 1b displays the CMC/MWCNT composites’ morphology still exhibits the tubular shape. The tube-walls are incredibly rough because of coated CMC on MWCNTs surface as well as the created composites twisted to one another, Some brittle and the cracks propagate in a planar fashion. Cellulose in the MWCNTs matrix was observed subsequently. A homogenous dispersion of MWCNT particles has been predominantly noted; the results recommend that cellulose has been grafted covalently upon MWCNTs’ surface, simplifying the MWCNTs dispersion in the CMC matrix, the cellulose matrix can be observed that the MWCNTs wrapped with CMC in a somewhat inconsistent and non-uniform manner. The ultrasonic procedure enhanced the development of MWCNT/CMC composite. The MWCNT/CMC composite has been investigated further by using TGA to find thermal stability along with their constituents (Figure 2). The weight loss curves of TGA for MWCNT/CMC composite exhibited three weight loss decay. In the starting stage, slightly weight loss among 50 °C to 100 °C has been noticed that was due to the moisture, loss of bound water, and dehydration of the composite.

The second-stage mass loss was in the range from 100 °C to 450 °C, and occured because of the depolymerization of cellulose and grafting of side chains on the surface of MWCNT [42]. At temperatures higher than 450 °C, he results show that near about 800 °C CNT weight loss began as well as demonstrated a high weight loss with a rise in temperature. This result signifies that the thermal stability of the sheet is improved by adding MWCNT [43,44,45].

**Figure 3:**
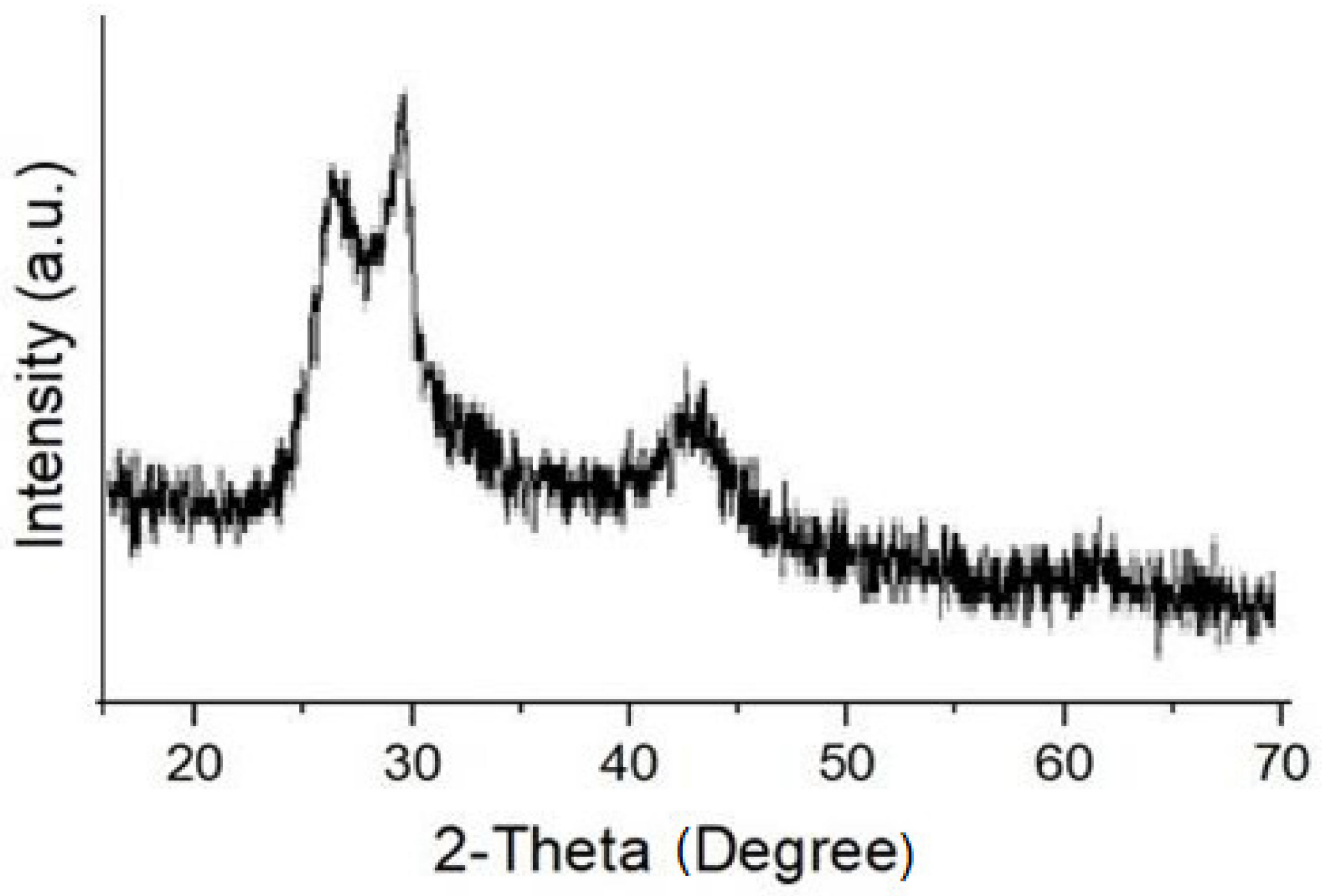
XRD spectra of MWCNT@celllulose nanocomposite showing degradation procedures.

Figure 3 shows the X-ray diffraction pattern of the MWCNT@celllulose nanocomposite. The appearance of peaks at 2*θ* = 16.5° and 30° could be attributed to the presence of cellulose, which is characteristic of the (200) plane refection of a graphite structure. This sharp peak of 30° indicates the neat arrangement of C atoms in the MWCNTs. The peak at 16.5° from (101) plane reflection was caused by the transverse arrangement of the crystallites in cellulose [46]. Peaks at 2*θ* = 26.6°, and 44.7° could be attributed to the characteristic peak of MWCNTs, indicating that the composite was successfully formed [47,48].

### 3.2. Electrocatalytic Oxidation of Amitriptyline at Multi-walled Carbon/Cellulose Nanocomposite

**Scheme 1:**
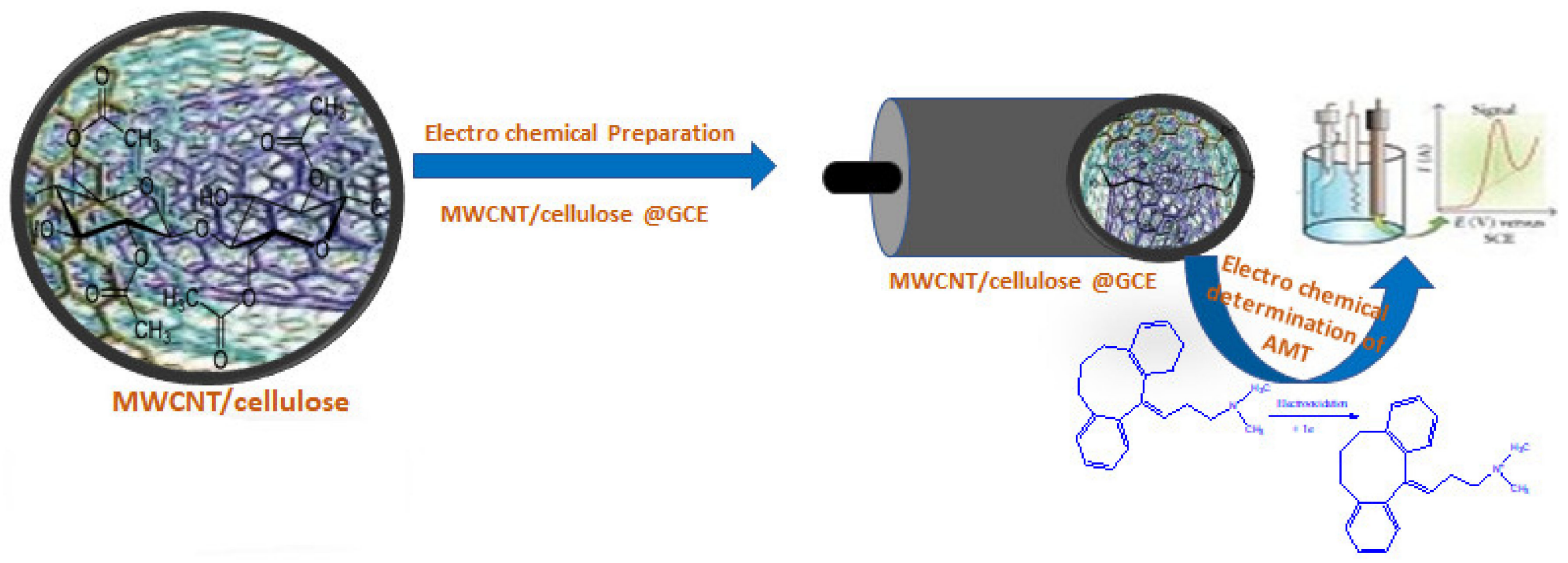
Schematic representation of electrocatalytic oxidation of amitriptyline (AMT) by utilizing of MWCNT/Cellulose/GCE and multi-walled carbon/cellulose nanocomposite.

A CV gives an amazing and helpful apparatus to figure out if an electrochemical response is diffusion or dynamically controlled. The oxidation of 2 mmol/L AMT by utilizing CV at an uncovered GCE was concentrated on over the potential sweep rates 50–250 mV/s. The desired active electrode of electrocatalytic oxidation of AMT has been set-up through GCE coated via a synthesized MWCNT/Cellulose nanocomposite (thin layer). The quality of binding among the GCE, along with NCs, has enhanced by the addition of 2 μL of a 2% glutaraldehyde aqueous solution. The assembled electrode has been determined electrocatalytic oxidation of AMT in a phosphate buffer medium with the utilization of electrochemical technique. The proposed electrocatalytic oxidation is shown with several advantages, such as long-term stability in the aqueous medium along with air, non-toxic nature, simply assemble, easy to handle, active electrochemical aspects, as well as chemical attributes. Scheme 1 demonstrates the mechanism of electrocatalytic oxidation of AMT with the MWCNT/Cellulose/GCE nanocomposite probe by electrochemical responses in the aqueous medium. Throughout the electrocatalytic oxidation performance of AMT determination, the applied electrochemical current is enhanced significantly on the surface of a thin film of MWCNT/Cellulose/GCE and simultaneously done the antibacterial activity of MWCNT/Cellulose nanocomposite.

### 3.3. Electrochemical Performance of AMT on MWCNT/Cellulose/GCE

**Figure 4:**
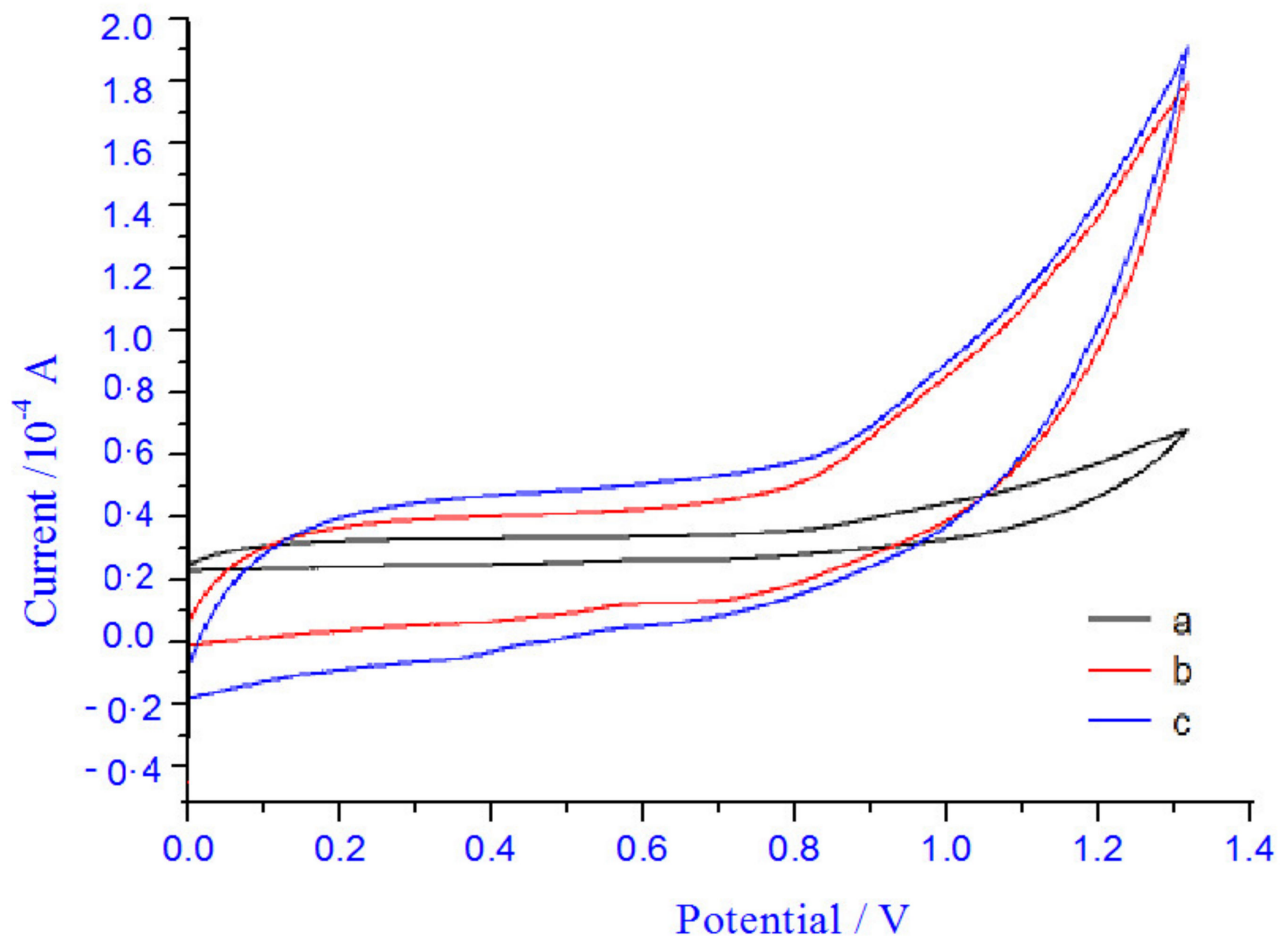
CV recorded at pH of 7.0 in 0.1 M PBS at a scan rate of 50 mVs^−1^ (**a**) blank CVs of MWCNT modified GCE (**b**) The bare CVs MWCNT/Cellulose/GCE (**c**) MWCNT/Cellulose/GCE in the presence of AMT.

The electrocatalysis measurement studies at different scan rates for the determination of AMT have been performed under given conditions. Figure 4 shows the electrochemical behavior of AMT at bare GCE as well as improved MWCNT/Cellulose/GCE electrodes have been studied by utilizing 0.1M PBS at pH 7.0 and scan rate 50 mVs^−1^.

Figure 4a shows that the current response of bare GCE in the absence of the AMT analyte has no characteristic peak. This indicates that the surface of the electrode is clean as well as free from fouling. The addition of 1 μM of AMT in PBS (0.1 M) as a supporting electrolyte at a sweep rate of 50 mVs^−1^ produced an irreversible anodic peak current of AML at 0.684 V (Figure 4b). The recorded signal improves as well as peak possibilities are shifted towards a detrimental benefit with increased present verifying the irreversible dynamics of the minimization procedure [49].

The electrochemical response of 1 μM of AMT in 0.1M of PBS as an electrolyte at MWCNT/Cellulose/GCE modified electrode (50 mVs^−1^ scan rate) for producing an irreversible anodic peak current of AMT at 0.765 V (Figure 4c). The anodic peak current of AMT at MWCNT/Cellulose/GCE modified electrode showed promoting effect as well as high stability to the AMT’s electrochemical oxidation. It was also noticed that the peak currents improved at the MWCNT/Cellulose/GCE electrode that gives more evidence to assert the MWCNT/Cellulose/GCE electrode possessed higher electrocatalytic activity to the AMT detection.

### 3.4. The Influence of pH of AMT on MWCNT/Cellulose/GCE

**Figure 5:**
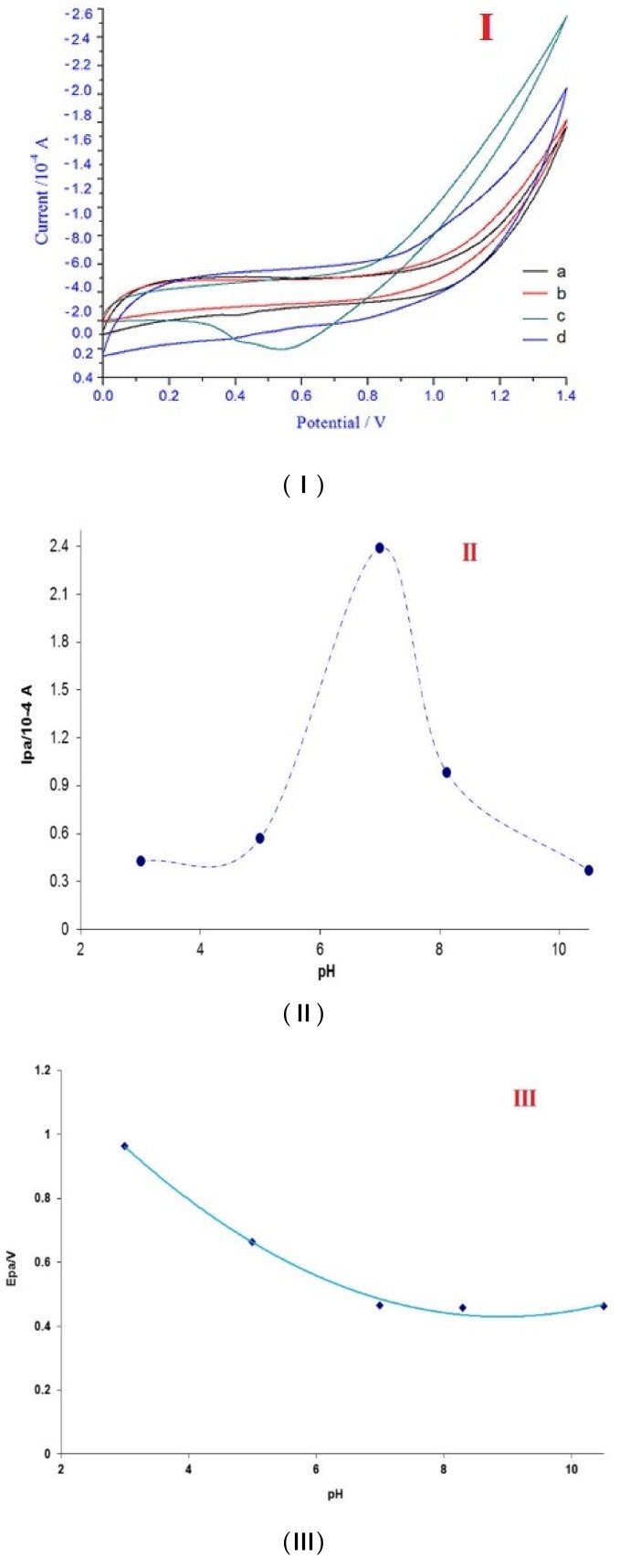
(**I**) Cyclic voltammograms at different pH on the shape of the anodic peaks in PBS solution at (**a**) 3.0, (**b**) 4.8, (**c**)7.0 and (**d**) pH 10.4 at 50 mVs^−1^. (**II**) Variation of currents with pH on GCE at scan rate of 50 mVs^−1^ in phosphate buffer (**III**) Influence of pH on the potential of AMT on GCE at a scan rate of 50 mVs^−1^ in phosphate buffer.

The electrocatalysis of AMT oxidation of pH was studied by the voltammetry method over 3.0-10.5 pH value in PBS solution (0.1M), which shows results in the inert electrolyte. Figure 5I shows plots of current (uA) versus potential V of the electrochemical reaction mixture of AMT. It was found that the MWCNT/Cellulose/GCE increasing negative potential with rising pH values suggesting the contribution of protons in the electrode reaction of AMT. Figure 5II shows a plotting graph of ipa vs. pH, and it was found that the oxidative anodic ipa of AMT increases with an increasing pH range of 6–7 pH and attains a maximum pH of 7.0. Therefore, the value of pH 7.0 has been picked up as the maximum pH for electrocatalysis of AMT oxidation in the MWCNT/Cellulose/GCE surface. After that, the peak current of the electrochemical reaction started decreasing slowly due to the deficiency of protons. Figure 4III The graph of anodic peak potential vs. pH has been plotted as well as expressed as *Epa(V) =* 0.0665 x *+* 1.0526 (*R =* 0.9848). Figure 5III shows obtained slope value in great accordance with the Nernstian worth of 0.059 V that implies the same quantity of electrons or protons in AMT’s oxidation.

**Scheme 2:**
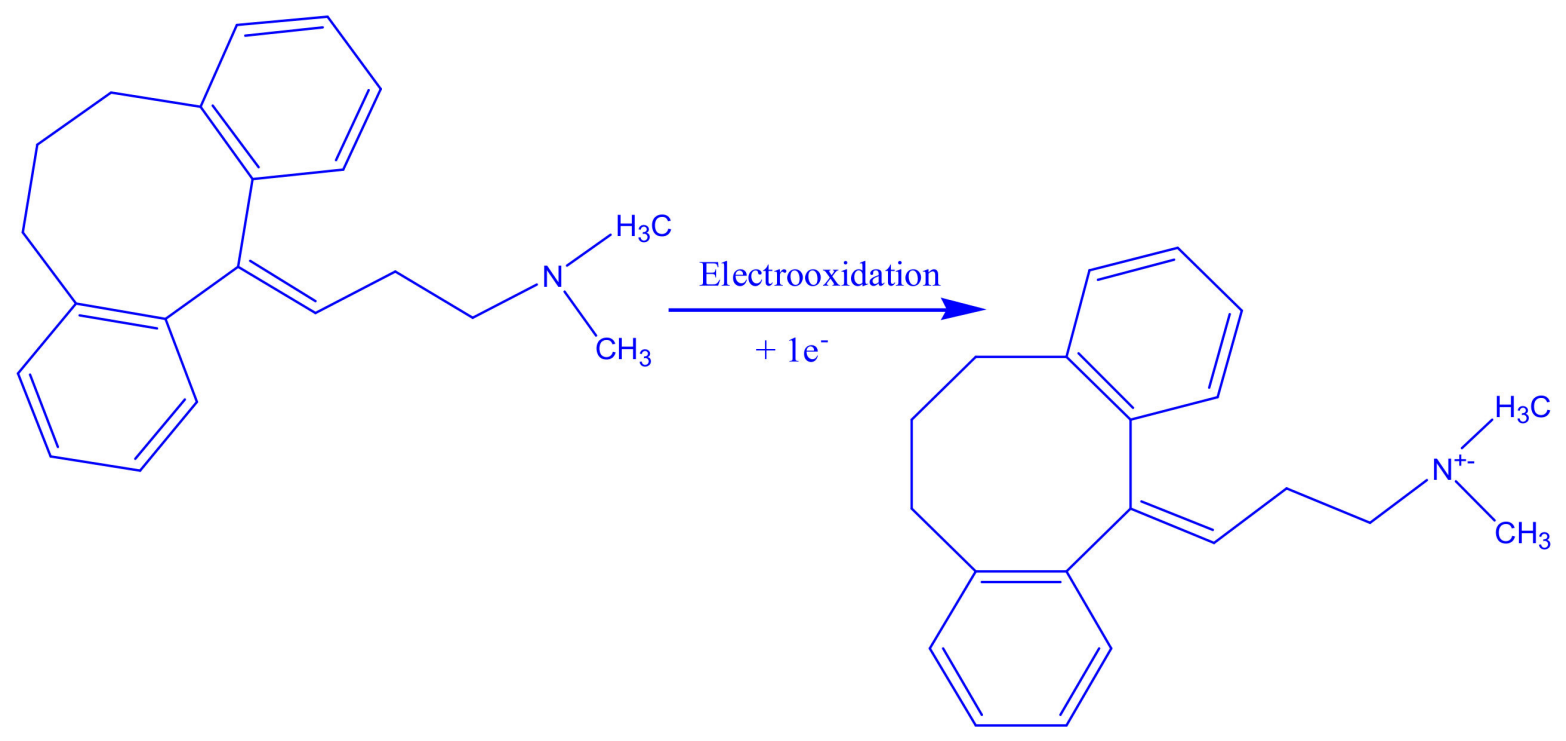
Proposed electrooxidation mechanism for AMT at the WCNT/Cellulose/GCE.

It was supposed that the oxidation of alkylamine nitrogen atoms entails the one-electron transfer that results in the development of extreme cations (oxidation mechanism) in Scheme 2. This particular procedure is facilitated by K_3_Fe(CN)_6,_ as it is recognized to become an oxidizing agent to obtain triethylamine radical cations [50]. Additionally, this mechanism has likewise been found for any electrochemical oxidation of aliphatic amines [51].

### 3.5. The Influence of Scan Rate on MWCNT/Cellulose/GCE for AMT

**Figure 6:**
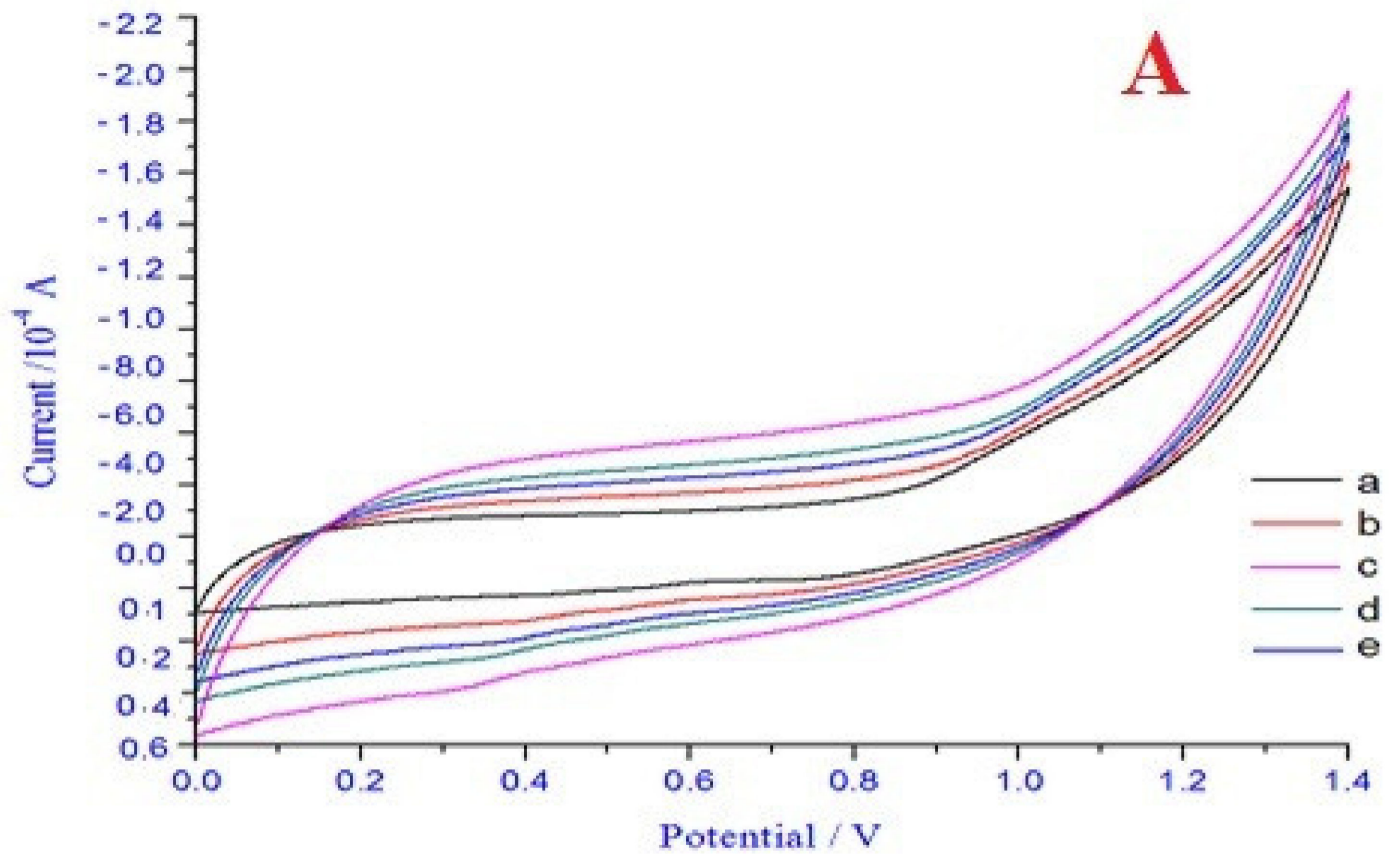

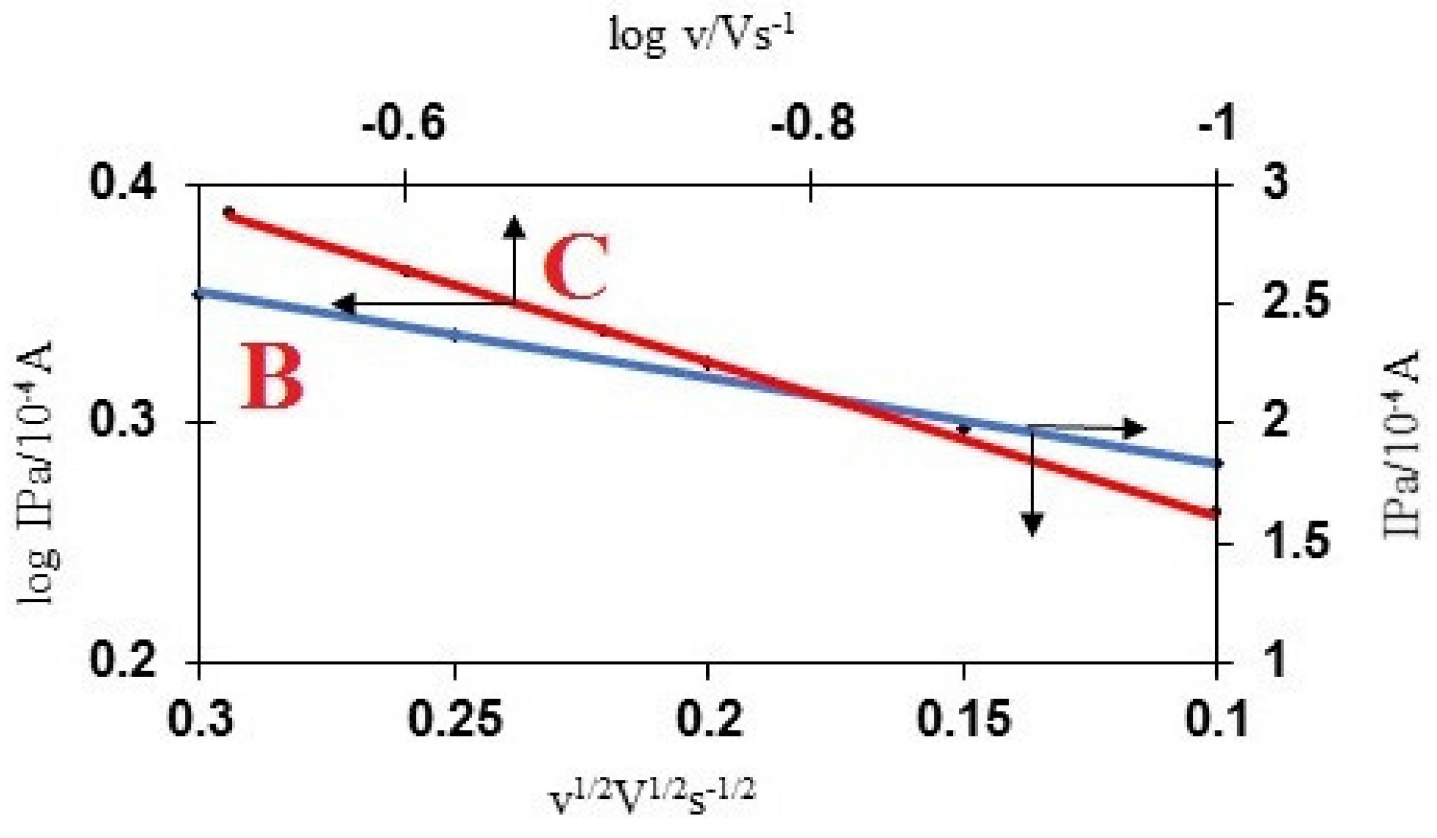
(**A**) Linear sweep voltammograms for the oxidation of AMT at a MWCNT/Cellulose/GCE with different scan rates of (**a**); 50 (**b**); 100 (**c**); 150 (**d**); 200 and (**e**) 250 mVs^−1^. (**B**–**C**) Dependence of the oxidation peak current vs scan rate square root and the logarithms of peak current vs scan rate.

The *MWCNT/Cellulose* modified electrode showed the CV responses at various scan rates of 50 mVs^−1^ to 250 mVs^−1^for 1mM of AMT in PBS (0.1 M) at neutral pH shown in Figure 6A. The obtained plot from a calibration curve was observed in the linearity regression equation Ipa (10^−4^ A) = 3.6218 *v*^1/2^ (V^1/2^ s^−1/2^) + 1.4753 (r = 0.9864) with the value of correlation coefficient 0.9956 in the range Figure 5B that shows a diffusion-controlled procedure occurring at the MWCNT/Cellulose modified electrode. In support of this, A calibration plot (log Ipa and log v) as it is in Figure 6C has been plotted as well to the equation of Ipa (10^−4^ A) = 0.2571 log *ν* (Vs^−1^) + 0.5188; (r^2^ = 0.9994). 0.25 Slope value has been compared with 0.5 theoretically expected value for a purely diffusion-controlled procedure [52,53], which confirms the AMT’s electrooxidation has been diffusion-controlled.

### 3.6. Validation Performance of MWCNT/Cellulose/GCE for AMT

To create a voltammetry way of identifying the AMT, the differential pulse voltammetry methods have been used underneath the enhanced experimental conditions. Five replicate dimensions have been captured at every concentration level. The outcomes are summarized in Table 1. The LOQ (quantification limit) along with LOD (detection limit) are main variables which characterize the functionality of an electrode determination at a low analyte concentration. Utilizing the DPV method, the feasibility of the suggested electrode just for the quantitative evaluation of AMT has been examined.

**Figure 7:**
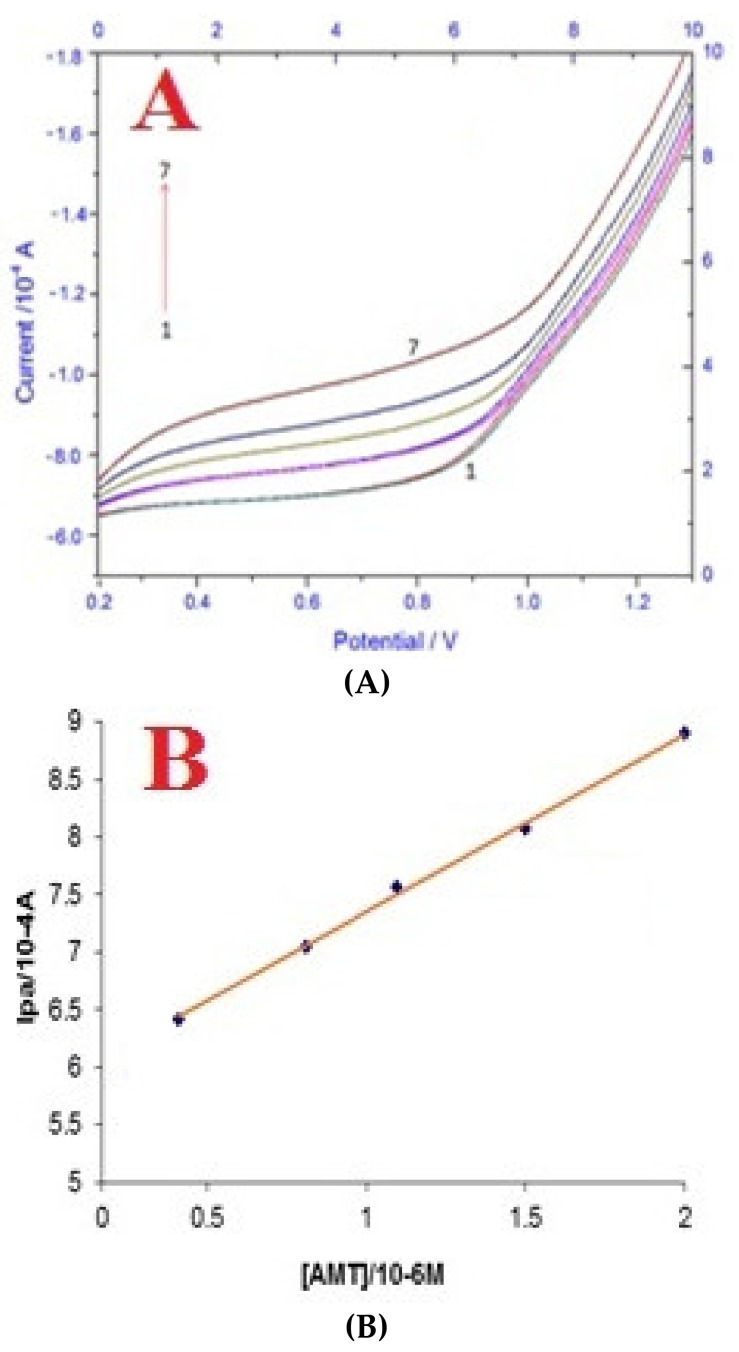
(**A**) Differential pulse voltammograms for increasing concentration of AMT (μM): (1) 0.5 (2) 7.5 (3) 10.0 (4) 15.0 (5) 20.0, other conditions are same as above. (**B**) Plot of current against the concentration of AMT.

A DP voltammogram has been found by adding AMT at pH 7.0 value in (0.1 M PBS). The peak current of AMT rise with rising concentration to 0.5–20μM, as shown in Figure 7A. Figure 7B shows the ipa vs. AMT plot from the calibration curve, the place that the regression of the linear situation is represented as Ipa = 2.36·x + 3.55 × 10^−2^ (R = 0.9985).

LOD was measured from a standard deviation of response and the calibration curve slope by using the LOD equation.

(1) LOD=3×S÷b

LOQ was measured dependent upon a standard deviation of the intercept as well as a calibration curve slope by utilizing the following equation.

(2) LOQ=10×S÷b,

where S is the standard deviation of five new experiments as well as b represents the calibration curve slope. The LOQ, along with LOD, had been determined to be 0.0845 μM along with 0.282 μM, respectively, from Eqs. (2) and (3). The results were (Table 1) indicate the good recoveries of the proposed method. Laboratory experiments further validated the calculated LOQ values of AMT, the MWCNT/Cellulose/GCE electrode demonstrated good recoveries as compared to previously utilized electrodes as mentioned in the literature by several researchers (see Table 2).

### 3.7. Application to Real Sample Analysis

DPV investigated the practical capability of the proposed technique to determine AMT in the human urine sample on MWCNT/Cellulose/GCE. Recovery researchers had been performed by spiking drug-free urine with knowing AMT amounts. To evaluate this method, the standard addition technique has been used by adding a known AMT amount in the urine sample solution. The graph based on the calibration of spiked samples was performed under optimized conditions for the determination of AMT at MWCNT/Cellulose/GCE. The results (Table 3) indicate good recoveries of the proposed technique. The mean recovery values were 98.6% to 100.4%, as well as the lower RSD values that show no interference of other active compounds present in this system (Table 3). It shows good accuracy as well as precision in the AMT’s analysis in real sample analysis of the proposed method.

## 4. Conclusions

In this study, a cheap and highly efficient MWCNT/Cellulose modified *GCE* electrode was prepared to oxidize AMT electrocatalytically in phosphate buffer solution under given physical condition at 7.0 pH value. The MWCNT/Cellulose/GCE electrode exhibited highly effective electrochemical oxidation performance for AMT. The oxidation of AMT has been obtained irreversible, having diffusion character. Suitable oxidation mechanisms were proposed. The peak at about 0.765V was suitable for analysis, and the peak current was linear to concentrations over a certain range under the selected conditions. This material can be utilized for voltammetry determination of selected analyte with good reproducibility of LOQ and LOD had been obtained. As a result, the study of influencing factors, repeatability and safety would help provide insight into the application of an electrochemical method for effective AMT in pharmaceutical formulations as well as human urine having (98.6% to 100.4%) good recoveries. The proposed method is suitable for quality control laboratories as well as pharmacokinetic studies where economy and time are essential.

## Figures and Tables

**Table 1:** Characteristics of AMT calibration plot using differential pulse voltammetry at glassy carbon electrode 0.5 to 20.0 μM.

Linearity range (μM) Slope of the calibration plot Intercept Correlation coefficient (r) RSD of slope ^a^ (%) RSD of intercept ^a^ (%) LOD (μM) LOQ (μM)	0.5–20.0 5.458 0.8207 0.9985 0.5812 1.5417 0.0845 0.282

^a^ Mean average of five determinations.

**Table 2:** of detection limits for AMT to different electrode modifiers.

Modifier	LOD	Ref.
Carbon nanotube CuO nanoparticles Montmorillonite nanoclay and ionic liquid CNT/SiO2/Al2O3/Nb2O5/DNA Multi-walledcarbon@celllulose nanocomposite	1.16 μM 0.4 μM 24 nM 0.12 μM 0.0845 μM	[54] [55] [56] [57] This work

**Table 3:** The application of MWCNT/Cellulose for determination of AMT in urine samples.

Sample	Spiked (μM)	Found ^a^ (μM)	Recovery (%)	Bias (%)	S.D. ± R.S.D (%)
1	0.5	0.502	100.4	0.40	0.021 ± 0.022
2	1.0	1.001	100.1	0.10	0.015 ± 0.011
3	1.5	1.481	98.70	−1.30	0.030 ± 0.016
4	2.0	1.972	98.60	−1.40	0.065 ± 0.032

^a^ Average of five determination.
